# An extension of the proximal point algorithm beyond convexity

**DOI:** 10.1007/s10898-021-01081-4

**Published:** 2021-09-06

**Authors:** Sorin-Mihai Grad, Felipe Lara

**Affiliations:** 1 Faculty of Mathematics, University of Vienna, Oskar-Morgenstern-Platz 1, A-1090 Vienna, Austria; 2 Departamento de Matemática, Facultad de Ciencias, Universidad de Tarapacá, Arica, Chile

**Keywords:** Nonsmooth optimization, Nonconvex optimization, Proximity operator, Proximal point algorithm, Generalized convex function

## Abstract

We introduce and investigate a new generalized convexity notion for functions called prox-convexity. The proximity operator of such a function is single-valued and firmly nonexpansive. We provide examples of (strongly) quasiconvex, weakly convex, and DC (difference of convex) functions that are prox-convex, however none of these classes fully contains the one of prox-convex functions or is included into it. We show that the classical proximal point algorithm remains convergent when the convexity of the proper lower semicontinuous function to be minimized is relaxed to prox-convexity.

## Introduction

The first motivation behind this study comes from works like [12, 19, 22, 23] where proximal point type methods for minimizing quasiconvex functions formulated by means of Bregman distances were proposed. On the other hand, other extensions of the proximal point algorithm for nonconvex optimization problems (such as the ones introduced in [10, 18, 20, 24]) cannot be employed in such situations due to various reasons. Looking for a way to reconcile these approaches we came across a new class of generalized convex functions that we called prox-convex, whose properties allowed us to extend the convergence of the classical proximal point algorithm beyond the convexity setting into a yet unexplored direction.

In contrast to other similar generalizations, the proximity operators of the proper prox-convex functions are single-valued (and firmly nonexpansive) on the underlying sets. To the best of our knowledge besides the convex and prox-convex functions only the weakly convex ones have single-valued proximity operators (cf. [16]). This property plays an important role in the construction of proximal point type algorithms as the new iterate is thus uniquely determined and does not have to be picked from a set. Moreover, the prox-convexity of the functions can be considered both globally or on a subset of their domains, that can be of advantage when dealing with concrete applications from practice. Various functions, among which several (strongly) quasiconvex, weakly and DC (i.e. difference of convex) ones, fulfill the definition of the new notion we propose. As a byproduct of our study we also deliver new results involving (strongly) quasiconvex functions.

Different to other extensions of the proximal point algorithm, the one we propose has a sort of a local nature, however not in the sense of properties of a function that hold in some neighborhoods, but concerning the restriction of the function to a (convex) set. We are not aware of very similar work in the literature where the proximity operator of a function is taken with respect to a given set, however in papers like [6, 13] such constructions with some employed functions not split from the corresponding sets were already considered.

Given a proper, lower semicontinuous and convex function $h: \mathbb {R}^{n} \rightarrow \overline{\mathbb {R}} := \mathbb {R} \cup \{\pm \infty \}$, for any $z \in \mathbb {R}^{n}$ the minimization problem

1.1 $$\begin{aligned} \min _{x \in \mathbb {R}^{n}} \left[ h(x) + \frac{1}{2} \Vert z - x \Vert ^{2}\right] \end{aligned}$$

has (even in more general frameworks such as Hilbert spaces) a unique optimal solution denoted by ${{\,\mathrm{Prox}\,}}_{h} (z)$, that is the value of the *proximity operator* of the function *h* at the point *z*. A fundamental property of the latter is when $z, \overline{x} \in \mathbb {R}^{n}$ (see, for instance, [5, Proposition 12.26])

1.2 $$\begin{aligned} \overline{x} = {{{\,\mathrm{Prox}\,}}}_{h} (z) ~ \Longleftrightarrow ~ z - \overline{x} \in \partial h(\overline{x}), \end{aligned}$$

where $\partial h$ is the usual convex subdifferential.

These two facts (the existence of an optimal solution to (1.1) and the characterization (1.2)) are crucial tools for proving the convergence of the proximal point type algorithms for continuous optimization problems consisting in minimizing (sums of) proper, lower semicontinuous and convex functions, and even for DC programming problems (see [4] for instance). For the class of prox-convex functions introduced in this article the first of them holds while the second one is replaced by a weaker variant and we show that these properties still guarantee the convergence of the sequence generated by the proximal point algorithm towards a minimum of a prox-convex function.

The paper is constructed as follows. After some preliminaries, where we define the framework and recall some necessary notions and results, we introduce and investigate the new classes of prox-convex functions and strongly G-subdifferentiable functions, showing that the proper and lower semicontinuous elements of the latter belong to the first one, too. Finally, we show that the classical proximal point algorithm can be extended to the prox-convex setting without losing the convergence.

## Preliminaries

By $\langle \cdot ,\cdot \rangle$ we mean the *inner product* of $\mathbb {R}^{n}$ and by $\Vert \cdot \Vert$ the Euclidean *norm* on $\mathbb {R}^{n}$. Let *K* be a nonempty set in $\mathbb {R}^{n}$ and we denote its *topological interior* by ${{\,\mathrm{int}\,}}K$ and its *boundary* by ${{\,\mathrm{bd}\,}}K$. The *indicator function* of *K* is defined by $\delta _{K} (x) := 0$ if $x \in K$, and $\delta _{K} (x):= + \infty$ elsewhere. By $\mathbb {B} (x, \delta )$ we mean the *closed ball* with center at $x\in \mathbb {R}^{n}$ and radius $\delta > 0$. By ${{\,\mathrm{Id}\,}}:\mathbb {R}^n\rightarrow \mathbb {R}^n$ we denote the *identity mapping* on $\mathbb {R}^n$.

Given any $x, y, z \in \mathbb {R}^{n}$, we have

2.1 $$\begin{aligned} \langle x - z, y - x \rangle = \frac{1}{2} \Vert z - y \Vert ^{2} - \frac{1}{2} \Vert x - z \Vert ^{2} - \frac{1}{2} \Vert y - x \Vert ^{2}. \end{aligned}$$

For any $x, y \in \mathbb {R}^{n}$ and any $\beta \in \mathbb {R}$, we have

2.2 $$\begin{aligned} \Vert \beta x + (1-\beta ) y \Vert ^{2} = \beta \Vert x \Vert ^{2} + (1 - \beta ) \Vert y\Vert ^{2} - \beta (1 - \beta ) \Vert x - y \Vert ^{2}. \end{aligned}$$

Given any extended-valued function $h:\mathbb {R}^{n}\rightarrow \overline{\mathbb {R}} := \mathbb {R}\cup \{ \pm \infty \}$, the *effective domain* of *h* is defined by ${{\,\mathrm{dom}\,}}\,h := \{x \in \mathbb {R}^{n}: h(x) < + \infty \}$. We say that *h* is *proper* if ${{\,\mathrm{dom}\,}}\,h$ is nonempty and $h(x) > - \infty$ for all $x \in \mathbb {R}^{n}$.

We denote by ${{\,\mathrm{epi}\,}}h := \{(x,t) \in \mathbb {R}^{n} \times \mathbb {R}: h(x) \le t\}$ the *epigraph* of *h*, by $S_{\lambda } (h) := \{x \in \mathbb {R}^{n}: h(x) \le \lambda \}$ (respectively $S^{<}_{\lambda } (h) := \{x \in \mathbb {R}^{n}: h(x) < \lambda \}$) the *sublevel* (respectively *strict sublevel*) *set* of *h* *at the height* $\lambda \in \mathbb {R}$, and by ${\arg \min }_{\mathbb {R}^{n}} h$ the set of all minimal points of *h*. We say that a function is *L-Lipschitz* when it is Lipschitz continuous with constant $L > 0$ on its domain. We adopt the usual conventions $\sup _{\emptyset } h := - \infty$, $\inf _{\emptyset } h := + \infty$ and $0(+\infty )=+\infty$.

A proper function *h* with a convex domain is said to be

(*a*): *convex* if, given any $x, y \in {{\,\mathrm{dom}\,}}\,h$, then
  2.3 $$\begin{aligned} h(\lambda x + (1-\lambda )y) \le \lambda h(x) + (1 - \lambda ) h(y), ~ \forall ~ \lambda \in [0, 1]; \end{aligned}$$
(*b*): *semistrictly quasiconvex* if, given any $x, y \in {{\,\mathrm{dom}\,}}~h,$ with $h(x) \ne h(y)$, then
  2.4 $$\begin{aligned} h(\lambda x + (1-\lambda )y) < \max \{h(x), h(y)\}, ~ \forall ~ \lambda \in \, ]0, 1[; \end{aligned}$$
(*c*): *quasiconvex* if, given any $x, y \in {{\,\mathrm{dom}\,}}\,h$, then
  2.5 $$\begin{aligned} h(\lambda x + (1-\lambda ) y) \le \max \{h(x), h(y)\}, ~ \forall ~ \lambda \in [0,1]. \end{aligned}$$
  We say that *h* is *strictly quasiconvex* if the inequality in (2.5) is strict (see [15, page 90]).

Every convex function is quasiconvex and semistrictly quasiconvex, and every semistrictly quasiconvex and lower semicontinuous function is quasiconvex (see [7, Theorem 2.3.2]). The function $h: \mathbb {R} \rightarrow \mathbb {R}$, with $h(x) := \min \{|x |, 1\}$, is quasiconvex, without being semistrictly quasiconvex.

A function *h* is said to be *neatly quasiconvex* (see [3, Definition 4.1]) if *h* is quasiconvex and for every $x\in \mathbb {R}^n$ with $h(x) > \inf h$, the sets $S_{h(x)} (h)$ and $S^{<}_{h(x)} (h)$ have the same closure (or equivalently, the same relative interior). As a consequence, a quasiconvex function *h* is neatly quasiconvex if and only if every local minimum of *h* is global minimum (see [3, Proposition 4.1]). In particular, every semistrictly quasiconvex function is neatly quasiconvex, and every continuous and neatly quasiconvex function is semistrictly quasiconvex by [3, Proposition 4.2]. The function in [3, Example 4.1] is neatly quasiconvex without being semistrictly quasiconvex. Recall that

$$\begin{aligned} h ~ \mathrm{is }\,\mathrm{convex}\Longleftrightarrow & {} {{\,\mathrm{epi}\,}}h ~ \mathrm{is} ~ \mathrm{a} ~ \mathrm{convex} ~ \mathrm{set;} \\ h ~ \mathrm{is ~ quasiconvex}\Longleftrightarrow & {} S_{\lambda } (h) ~ \mathrm{is ~ a ~ convex ~ set ~ for ~ all ~ } \lambda \in \mathbb {R}. \end{aligned}$$

For algorithmic purposes, the following notions from [5, Definition 10.27] (see also [29, 30]) are useful.

A function *h* with a convex domain is said to be *strongly convex* (respectively *strongly quasiconvex*), if there exists $\beta \in ]0, + \infty [$ such that for all $x, y \in {{\,\mathrm{dom}\,}}\,h$ and all $\lambda \in [0, 1]$, we have

2.6 $$\begin{aligned}&h(\lambda y + (1-\lambda )x) \le \lambda h(y) + (1-\lambda ) h(x) - \lambda (1 - \lambda ) \frac{\beta }{2} \Vert x - y \Vert ^{2}, \end{aligned}$$

2.7 $$\begin{aligned}&\left( \mathrm{respectively} ~ h(\lambda y + (1-\lambda )x) \le \max \left\{ h(y), h(x)\right\} - \lambda (1 - \lambda ) \frac{\beta }{2} \Vert x - y \Vert ^{2}. \right) \end{aligned}$$

For (2.7), sometimes one needs to restrict the value $\beta$ to a subset *J* in $]0, + \infty [$ and then *h* is said to be *strongly quasiconvex on J*.

Every strongly convex function is strongly quasiconvex, and every strongly quasiconvex function is semistrictly quasiconvex. Furthermore, a strongly quasiconvex function has at most one minimizer on a convex set $K \subseteq \mathbb {R}^n$ that touches its domain (see [5, Proposition 11.8]).

A function $h: \mathbb {R}^{n} \rightarrow \overline{\mathbb {R}}$ is said to be

(*a*): *supercoercive* if
  2.8 $$\begin{aligned} \liminf _{\Vert x \Vert \rightarrow + \infty } \frac{h(x)}{\Vert x \Vert } = + \infty ; \end{aligned}$$
(*b*): *coercive* if
  2.9 $$\begin{aligned} \lim _{\Vert x \Vert \rightarrow + \infty } h(x) = + \infty ; \end{aligned}$$
(*c*): *weakly coercive* if
  2.10 $$\begin{aligned} \liminf _{\Vert x \Vert \rightarrow + \infty } \frac{h(x)}{\Vert x \Vert } \ge 0; \end{aligned}$$
(*d*): 2*-weakly coercive* if
  2.11 $$\begin{aligned} \liminf _{\Vert x \Vert \rightarrow + \infty } \frac{h(x)}{\Vert x \Vert ^{2}} \ge 0. \end{aligned}$$

Clearly, $(a) \Rightarrow (b) \Rightarrow (c) \Rightarrow (d)$. The function $h(x) = \sqrt{|x |}$ is coercive without being supercoercive; the function $h(x) = - \sqrt{|x |}$ is weakly coercive without being coercive (moreover, it is not even bounded from below). Finally, the function $h(x) = - |x |$ is 2-weakly coercive without being weakly coercive. Recall that *h* is coercive if and only if $S_{\lambda } (h)$ is a bounded set for every $\lambda \in \mathbb {R}$. A survey on coercivity notions is [8].

The *convex subdifferential* of a proper function $h: \mathbb {R}^{n} \rightarrow \overline{\mathbb {R}}$ at $x \in \mathbb {R}^{n}$ is

2.12 $$\begin{aligned} \partial h(x) := \left\{ \xi \in \mathbb {R}^{n}: h(y) \ge h(x) + \langle \xi , y - x \rangle , ~ \forall ~ y \in \mathbb {R}^{n}\right\} , \end{aligned}$$

when $x \in {{\,\mathrm{dom}\,}}\,h$, and $\partial h(x) = \emptyset$ if $x \not \in {{\,\mathrm{dom}\,}}\,h$. But in case of nonconvex functions (quasiconvex for instance) the convex subdifferential is too small and often empty, other subdifferential notions (see [14, 25]) being necessary, like the *Gutiérrez subdifferential* (of *h* at *x*), defined by

2.13 $$\begin{aligned} \partial ^{\le } h(x) := \left\{ \xi \in \mathbb {R}^{n}: ~ h(y) \ge h(x) + \langle \xi , y - x \rangle , ~ \forall ~ y \in S_{h(x)} (h)\right\} , \end{aligned}$$

when $x \in {{\,\mathrm{dom}\,}}\,h$, and $\partial ^{\le } h(x) = \emptyset$ if $x \not \in {{\,\mathrm{dom}\,}}\,h$, or the *Plastria subdifferential* (of *h* at *x*), that is

2.14 $$\begin{aligned} \partial ^{<} h(x) := \left\{ \xi \in \mathbb {R}^{n}: ~ h(y) \ge h(x) + \langle \xi , y - x \rangle , ~ \forall ~ y \in S^{<}_{h(x)} (h)\right\} , \end{aligned}$$

when $x \in {{\,\mathrm{dom}\,}}\,h$, and $\partial ^{<} h(x) = \emptyset$ if $x \not \in {{\,\mathrm{dom}\,}}\,h$. Clearly, $\partial h \subseteq \partial ^{\le } h \subseteq \partial ^{<} h$. The reverse inclusions do not hold as the function $h: \mathbb {R} \rightarrow \mathbb {R}$ given by $h(x) = \min \{x, \max \{x-1, 0\}\}$ shows (see [26, page 21]). A sufficient condition for equality in this inclusion chain is given in [26, Proposition 10].

Note that both $\partial ^{\le } h$ and $\partial ^{<} h$ are (at any point) either empty or unbounded, and it holds (see [14, 25, 26])

2.15 $$\begin{aligned} 0 \in \partial ^{<} h(x) \Longleftrightarrow 0 \in \partial ^{\le } h(x) \Longleftrightarrow x \in \arg \min _{\mathbb {R}^{n}}\,h \Longleftrightarrow \partial ^{<} h(x) = \mathbb {R}^{n}. \end{aligned}$$

However, one may have $\partial ^{\le } h(x) \ne \mathbb {R}^{n}$ at some minimizer of *h*.

We recall the following results originally given in [25, Theorem 2.3], [31, Proposition 2.5 and Proposition 2.6] and [9, Theorem 20], respectively.

### Lemma 2.1

Let $h: \mathbb {R}^{n} \rightarrow \overline{\mathbb {R}}$ be a proper function. The following results hold.

(*a*): If *h* is quasiconvex and *L*-Lipschitz, then $\partial ^{<} h(x) \ne \emptyset$ for all $x \in \mathbb {R}^{n}$. Moreover, there exists $\xi \in \partial ^{<} h(x)$ such that $\Vert \xi \Vert \le L$.
(*b*): If *h* is neatly quasiconvex and *L*-Lipschitz, then $\partial ^{ \le } h (x) \ne \emptyset$ for all $x \in \mathbb {R}^{n}$. Moreover, if $u \in \partial ^{\le } h(x)$, $u \ne 0$, then $L \frac{1}{\Vert u \Vert }u \in \partial ^{\le } h(x)$.

For $\gamma >0$ we define the *Moreau envelope of parameter* ${\gamma }$ of *h* by

2.16 $$\begin{aligned} ^{\gamma }h(z) = \inf _{x \in \mathbb {R}^{n}} \left( h(x) + \frac{1}{2\gamma } \Vert z-x \Vert ^{2}\right) . \end{aligned}$$

The *proximity operator of parameter* $\gamma >0$ of a function $h:\mathbb {R}^{n} \rightarrow \overline{\mathbb {R}}$ at $x \in \mathbb {R}^{n}$ is defined as

2.17 $$\begin{aligned} {{{\,\mathrm{Prox}\,}}}_{\gamma h}: \mathbb {R}^{n} \rightrightarrows \mathbb {R}^{n},\ {{{\,\mathrm{Prox}\,}}}_{\gamma h}(x)= \arg \min \limits _{y \in \mathbb {R}^{n} } \left\{ h(y) + \frac{1}{2 \gamma } \Vert y-x\Vert ^2\right\} . \end{aligned}$$

When *h* is proper, convex and lower semicontinuous, ${{{\,\mathrm{Prox}\,}}}_{\gamma h}$ turns out to be a single-valued operator. By a slight abuse of notation, when ${{{\,\mathrm{Prox}\,}}}_{\gamma h}$ is single-valued we write in this paper ${{{\,\mathrm{Prox}\,}}}_{\gamma h}(z)$ (for some $z\in \mathbb {R}^n$) to identify the unique element of the actual set ${{{\,\mathrm{Prox}\,}}}_{\gamma h}(z)$. Moreover, when $\gamma =1$ we write ${{{\,\mathrm{Prox}\,}}}_{h}$ instead of ${{{\,\mathrm{Prox}\,}}}_{1 h}$.

For studying constrained optimization problems, the use of properties restricted to some sets becomes important since they ask for weaker conditions. Indeed, for instance, the function $h: \mathbb {R} \rightarrow \mathbb {R}$ given by $h(x) = \min \{|x |, 2\}$ is convex on $K = [-2, 2]$, but is not convex on $\mathbb {R}$.

For a nonempty set *K* in $\mathbb {R}^{n}$, by $\partial _{K} h(x), \partial ^{\le }_{K} h(x)$ and $\partial ^{<}_{K} h(x)$, we mean the convex, Gutiérrez and Plastria subdifferentials of *h* at $x \in K$ restricted to the set *K*, that is,

$$\begin{aligned} \partial _{K} h(x) := \partial \left( h+\delta _K\right) (x) = \left\{ \xi \in \mathbb {R}^{n}: ~ h(y) \ge h(x) + \langle \xi , y - x \rangle , ~ \forall ~ y \in K \right\} , \end{aligned}$$

as well as $\partial ^{\le }_{K} h(x) := \partial ^{\le } (h+\delta _K)(x)$ and $\partial ^{<}_{K} h(x) := \partial ^{<} (h+\delta _K)(x)$.

For $K\subseteq \mathbb {R}^{n}$, a single-valued operator $T: K \rightarrow \mathbb {R}^{n}$ is called

(*a*): *monotone* on *K*, if for all $x, y \in K$, we have
  2.18 $$\begin{aligned} \langle T(x) - T(y), x - y \rangle \ge 0; \end{aligned}$$
(*b*): *firmly nonexpansive* if for every $x, y \in K$, we have
  2.19 $$\begin{aligned} \Vert T(x) - T(y) \Vert ^{2} + \Vert ({{\,\mathrm{Id}\,}}- T) (x) - ({{\,\mathrm{Id}\,}}- T) (y) \Vert ^{2} \le \Vert x - y \Vert ^{2}, ~ \forall ~ x, y \in K, \end{aligned}$$

According to [5, Proposition 4.4], *T* is firmly nonexpansive if and only if

2.20 $$\begin{aligned} \Vert T(x) - T(y) \Vert ^{2} \le \langle x - y, T(x) - T(y) \rangle , ~ \forall ~ x, y \in K. \end{aligned}$$

As a consequence, if *T* is firmly nonexpansive, then *T* is Lipschitz continuous and monotone.

## Prox-convex functions

In this section, we introduce and study a class of functions for which the necessary fundamental properties presented in the introduction are satisfied.

### Motivation, definition and basic properties

We begin with the following result, in which we provide a general sufficient condition for the nonemptiness of the values of the proximity operator.

#### Proposition 3.1

Let $h: \mathbb {R}^{n} \rightarrow \overline{\mathbb {R}}$ be a proper, lower semicontinuous and 2-weakly coercive function. Given any $z \in \mathbb {R}^{n}$, there exists $\overline{x} \in {{\,\mathrm{Prox}\,}}_{h} (z)$.

#### Proof

Given $z \in \mathbb {R}^{n}$, we consider the minimization problem:

3.1 $$\begin{aligned} \min _{x \in \mathbb {R}^{n}} h_{z} (x) := h(x) + \frac{1}{2} \Vert x - z \Vert ^{2}. \end{aligned}$$

Since *h* is lower semicontinuous and 2-weakly coercive, $h_{z}$ is lower semicontinuous and coercive by [8, Theorem 2(*ii*)]. Thus, there exists $\overline{x} \in \mathbb {R}^{n}$ such that $\overline{x} \in {\arg \min }_{\mathbb {R}^{n}} h_{z}$, i.e., $\overline{x} \in {{\,\mathrm{Prox}\,}}_{h} (z)$. $\square$

One cannot weaken the assumptions of Proposition 3.1 without losing its conclusion.

#### Remark 3.1

(*i*): Note that every convex function is 2-weakly coercive, and every bounded from below function is also 2-weakly coercive. The function $h: \mathbb {R}^{n} \rightarrow \mathbb {R}$ given by $h(x) = - |x |$ is 2-weakly coercive, but is neither convex nor bounded from below. However, for any $z \in \mathbb {R}^{n}$, ${{\,\mathrm{Prox}\,}}_{h} (z) \ne \emptyset$.
(*ii*): The 2-weak coercivity assumption can not be dropped in the general case. Indeed, the function $h: \mathbb {R} \rightarrow \mathbb {R}$ given by $h(x) = -x^{3}$ is continuous and quasiconvex, but fails to be 2-weakly coercive and for any $z \in \mathbb {R}$ one has ${{\,\mathrm{Prox}\,}}_{h} (z) = \emptyset$.

Next we characterize the existence of solution in the definition of the proximity operator.

#### Proposition 3.2

Let $h: \mathbb {R}^{n} \rightarrow \overline{\mathbb {R}}$ be a proper function. Given any $z \in \mathbb {R}^{n}$, one has

3.2 $$\begin{aligned} \overline{x} \in {{{\,\mathrm{Prox}\,}}}_{h} (z) ~ \Longleftrightarrow ~ h(\overline{x}) - h(x) \le \frac{1}{2} \langle \overline{x} + x - 2z, x - \overline{x} \rangle , ~ \forall x \in \mathbb {R}^{n}. \end{aligned}$$

#### Proof

Let $z\in \mathbb {R}^{n}$. One has

$$\begin{aligned} \overline{x} \in {{{\,\mathrm{Prox}\,}}}_{h} (z)&\Longleftrightarrow \, h(\overline{x}) + \frac{1}{2} \Vert \overline{x} - z \Vert ^{2} \le h(x) + \frac{1}{2} \Vert x - z \Vert ^{2}\ \forall \, x \in \mathbb {R}^{n} \\&\Longleftrightarrow \, h(\overline{x}) - h(x) \le \frac{1}{2} \Vert x - z \Vert ^{2} - \frac{1}{2} \Vert \overline{x} - z \Vert ^{2} \ \forall \, x \in \mathbb {R}^{n} \\&\Longleftrightarrow \, h(\overline{x}) - h(x) \le \langle \overline{x} - z, x - \overline{x} \rangle + \frac{1}{2} \Vert x - \overline{x} \Vert ^{2} \ \forall \, x \in \mathbb {R}^{n} \\&\Longleftrightarrow \, h(\overline{x}) - h(x) \le \frac{1}{2} \langle \overline{x} + x - 2z, x - \overline{x} \rangle \ \forall \, x \in \mathbb {R}^{n}. \end{aligned}$$

$$\square $$

Relation (3.2) is too general for providing convergence results for proximal point type algorithms while relation (1.2) has proven to be extremely useful in the convex case. Motivated by this, we introduce the class of *prox-convex functions* below. In the following, we write

3.3 $$\begin{aligned} {{{\,\mathrm{Prox}\,}}}_{h} (K, z) := {{{\,\mathrm{Prox}\,}}}_{\left( h + \delta _{K}\right) } (z). \end{aligned}$$

Note that closed formulae for the proximity operator of a sum of functions in terms of the proximity operators of the involved functions are known only in the convex case and under demanding hypotheses, see, for instance, [1]. However, constructions like the one in (3.3) can be found in the literature on proximal point methods for solving different classes of (nonconvex) optimization problems, take for instance [6, 13].

#### Definition 3.1

Let *K* be a closed set in $\mathbb {R}^{n}$ and $h: \mathbb {R}^{n} \rightarrow \overline{\mathbb {R}}$ be a proper function such that $K \cap {{\,\mathrm{dom}\,}}\,h \ne \emptyset$. We say that *h* is *prox-convex on* *K* if there exists $\alpha > 0$ such that for every $z \in K$, ${{\,\mathrm{Prox}\,}}_{h} (K, z) \ne \emptyset$, and

3.4 $$\begin{aligned} \overline{x} \in {{{\,\mathrm{Prox}\,}}}_{h} (K, z) ~ \Longrightarrow ~ h(\overline{x}) - h(x) \le \alpha \langle \overline{x} - z, x - \overline{x} \rangle , ~ \forall x \in K. \end{aligned}$$

The set of all prox-convex function on *K* is denoted by $\Phi (K)$, and the scalar $\alpha > 0$ for which (3.4) holds is said to be the *prox-convex value* of the function *h* on *K*. When $K=\mathbb {R}^{n}$ we say that *h* is *prox-convex*.

#### Remark 3.2

(*i*): One can immediately notice that $\overline{x} \in {{{\,\mathrm{Prox}\,}}}_{h} (K, z)$ (from (3.4)) yields $\overline{x} \in K\cap {{\,\mathrm{dom}\,}}\,h$, and, on the other hand, (3.4) is equivalent to a weaker version of (1.2), namely
  $$\begin{aligned} \overline{x} \in {{{\,\mathrm{Prox}\,}}}_{h} (K, z) ~ \Longrightarrow ~ z - \overline{x} \in \partial \left( \frac{1}{\alpha } \left( h+ \delta _K\right) \right) (\overline{x}). \end{aligned}$$
(*ii*): The scalar $\alpha > 0$ for which (3.4) holds needs not be unique. Indeed, if *h* is convex, then $\alpha = 1$ by Proposition 3.4. However, due to the convexity of *h*, $\langle \overline{x} - z, x - \overline{x} \rangle \ge 0$. Hence, $\overline{x} \in {{\,\mathrm{Prox}\,}}_{h} (K, z)$ implies that
  $$\begin{aligned} h(\overline{x}) - h(x) \le \langle \overline{x} - z, x - \overline{x} \rangle \le \gamma \langle \overline{x} - z, x - \overline{x} \rangle , ~ \forall ~ \gamma \ge 1,\ ~ \forall ~ x \in K. \end{aligned}$$
  Note however that a similar result does not necessarily hold in general, as $\langle \overline{x} - z, x - \overline{x} \rangle$ might be negative.
(*iii*): Note also that, at least from the computational point of view, an exact value of $\alpha$ needs not be known, as one can see in Sect. 4.

In the following statement we see that in the left-hand side of (3.4) one can replace the element-of symbol with equality since the proximity operator of a proper prox-convex function is single-valued and also firmly nonexpansive.

#### Proposition 3.3

Let *K* be a closed set in $\mathbb {R}^{n}$ and $h: \mathbb {R}^{n} \rightarrow \overline{\mathbb {R}}$ a proper prox-convex function on *K* such that $K \cap {{\,\mathrm{dom}\,}}\,h \ne \emptyset$. Then the map $z \rightarrow {{\,\mathrm{Prox}\,}}_{h} (K, z)$ is single-valued and firmly nonexpansive on *K*.

#### Proof

Suppose that *h* is a prox-convex function with prox-convex value $\alpha > 0$ and assume that for some $z\in K$ one has $\{\overline{x}_{1}, \overline{x}_{2}\} \subseteq {{\,\mathrm{Prox}\,}}_{h} (K, z)$. Then

3.5 $$\begin{aligned}&h\left( \overline{x}_{1}\right) - h(x) \le \alpha \langle \overline{x}_{1} - z, x - \overline{x}_{1} \rangle , ~ \forall x \in K, \end{aligned}$$

3.6 $$\begin{aligned}&h\left( \overline{x}_{2}\right) - h(x) \le \alpha \langle \overline{x}_{2} - z, x - \overline{x}_{2} \rangle , ~ \forall x \in K. \end{aligned}$$

Take $x = \overline{x}_{2}$ in (3.5) and $x = \overline{x}_{1}$ in (3.6). By adding the resulting equations, we get

$$\begin{aligned} 0 \le \alpha \langle \overline{x}_{1} - \overline{x}_{2}, \overline{x}_{2} - z + z -\overline{x}_{1} \rangle = -\alpha \Vert \overline{x}_{1} - \overline{x}_{2}\Vert ^2 \le 0. \end{aligned}$$

Hence, $\overline{x}_{1} = \overline{x}_{2}$, consequently ${{\,\mathrm{Prox}\,}}_{h} (K, \cdot )$ is single-valued.

Let $z_{1}, z_{2} \in K$ and take $\overline{x}_{1} \in {{\,\mathrm{Prox}\,}}_{h} (K, z_{1})$ and $\overline{x}_{2} \in {{\,\mathrm{Prox}\,}}_{h} (K, z_{2})$. One has

3.7 $$\begin{aligned}&h\left( \overline{x}_{1}\right) - h(x) \le \alpha \langle \overline{x}_{1} - z_{1}, x - \overline{x}_{1} \rangle , ~ \forall ~ x \in K, \end{aligned}$$

3.8 $$\begin{aligned}&h\left( \overline{x}_{2}\right) - h(x) \le \alpha \langle \overline{x}_{2} - z_{2}, x - \overline{x}_{2} \rangle , ~ \forall ~ x \in K. \end{aligned}$$

Taking $x = \overline{x}_{2}$ in (3.7) and $x = \overline{x}_{1}$ in (3.8) and adding them, we have

$$\begin{aligned} \Vert \overline{x}_{1} - \overline{x}_{2} \Vert ^{2} \le \langle z_{1} - z_{2}, \overline{x}_{1} - \overline{x}_{2} \rangle . \end{aligned}$$

Hence, by [5, Proposition 4.4], ${{\,\mathrm{Prox}\,}}_{h} (K, \cdot )$ is firmly nonexpansive. $\square$

Next we show that every lower semicontinuous and convex function is prox-convex.

#### Proposition 3.4

Let *K* be a closed and convex set in $\mathbb {R}^{n}$ and $h:\mathbb {R}^{n} \rightarrow \overline{\mathbb {R}}$ be a proper and lower semicontinuous function such that $K \cap {{\,\mathrm{dom}\,}}\,h \ne \emptyset$. If *h* is convex on *K*, then $h \in \Phi (K)$ with $\alpha =1$.

#### Proof

Since *h* is convex, the function $x \mapsto h(x) + ({\beta }/{2}) \Vert z - x \Vert ^{2}$ is strongly convex on *K* for all $\beta > 0$ and all $z \in K$, in particular, for $\beta = 1$. Thus ${{\,\mathrm{Prox}\,}}_{h} (K, z)$ contains exactly one element, say $\overline{x} \in \mathbb {R}^{n}$. It follows from [5, Proposition 12.26] that $z - \overline{x} \in \partial (h + \delta _K) (\overline{x})$, so relation (3.4) holds for $\alpha = 1$. Therefore, $h \in \Phi (K)$. $\square$

Prox-convexity goes beyond convexity as shown below.

#### Example 3.1

Let us consider $K := [0, 1]$ and the continuous and real-valued function $h: \mathbb {R} \rightarrow \mathbb {R}$ given by $h(x) = - x^{2} - x$. Note that

(*i*): *h* is strongly quasiconvex on *K* without being convex (take $\beta = 1$);
(*ii*): For all $z \in K$, ${{\,\mathrm{Prox}\,}}_{h} (K, z) = {\arg \min }_{K} h = \{1\}$;
(*iii*): $\partial ^{\le }_{K} h(1) = K$ since, by (*ii*), $K \cap S_{h(1)} (h) = \{1\}$, i.e., $\partial ^{\le }_{K} h(1) = K$ by (2.15);
(*iv*): *h* satisfies condition (3.4) for all $\alpha \in \, ]0, 2]$. Indeed, for all $z \in K \backslash \{1\}$, ${{\,\mathrm{Prox}\,}}_{h} (K, z) = {\arg \min }_{K} h = \{1\}$, thus the right-hand side of (3.4) turns into $-2 + x^{2} + x \le \alpha (1-z)(x-1)$ for all $x \in [0, 1]$, that is further equivalent to $(x+2) \ge \alpha (1-z)$ for all $x \in [0, 1]$, and then to
  $$\begin{aligned} \alpha \le \frac{x + 2}{1 - z} = \frac{x}{1 - z} + \frac{2}{1 - z} ~ \forall ~ x \in [0, 1]. \end{aligned}$$
  The last inequality is fulfilled for all $x, z \in [0, 1]$ with $z \ne 1$ when $\alpha \in \, ]0, 2]$.
(*v*): $$h \in \Phi (K)$$.

In order to formulate a reverse statement of Proposition 3.4, we note that if $h:\mathbb {R}^{n}\rightarrow \overline{\mathbb {R}}$ is a lower semicontinuous and prox-convex function on some set $K \cap {{\,\mathrm{dom}\,}}\,h \ne \emptyset$ which satisfies (3.4) for $\alpha = 1$, then *h* is not necessarily convex. Indeed, the function in Example 3.1 satisfies (3.4) for all $\alpha \in \, ]0, 2]$, but it is not convex on $K = [0, 1]$.

In the following example, we show that lower semicontinuity is not a necessary condition for prox-convexity. Note also that although the proximity operator of the function mentioned in Remark 3.1(*ii*) is always empty, this is no longer the case when restricting it to an interval.

#### Example 3.2

Take $n \ge 3$, $K_{n} := [1, n]$ and the function $h_{n}: K_{n} \rightarrow \mathbb {R}$ given by

$$\begin{aligned} h_{n} (x) = \left\{ \begin{array}[c]{cl} 1- x^{3}, &{} \mathrm {if} ~ 1 \le x \le 2, \\ 1- x^{3} - k, &{} \mathrm {if} ~ k < x \le k+1, ~ k \in \{2, \ldots , n-1\}. \end{array} \right. \end{aligned}$$

Note that $h_{n}$ is neither convex nor lower semicontinuous, but it is quasiconvex on $K_{n}$. Due to the discontinuity of $h_{n}$, the function $f_{n} (x) = h_{n} (x) + ({1}/{2}) \Vert x \Vert ^{2}$ is neither convex nor lower semicontinuous on $K_{n}$, hence $h_{n}$ is not *c*-weakly convex (in the sense of [17]) either and also its subdifferential is not hypomonotone (as defined in [10, 18, 24]). However, for any $z \in K_{n}$, ${{\,\mathrm{Prox}\,}}_{h_{n}} (K_n, z) = \{n\}$, and $\partial ^{\le }_{K_{n}} h_{n} (n) = K_{n}$. Therefore, $h_{n} \in \Phi (K_{n})$.

Another example of a prox-convex function that is actually (like the one in Example 3.1) both concave and DC follows.

#### Example 3.3

Take $K=[1, 2]$ and $h: ]0, + \infty [ \rightarrow \mathbb {R}$ defined by $h(x)= 5x + \ln (1+10x)$. As specified in [21], both the prox-convex function presented in Example 3.1 and this one represent cost functions considered in oligopolistic equilibrium problems, being thus relevant for studying also from a practical point of view. One can show that ${{\,\mathrm{Prox}\,}}_{h} (K, z) = {\arg \min }_{K} h = \{1\}$ for all $z \in K$ and (3.4) is fulfilled for $\alpha \in \, ]0, 5[$.

#### Remark 3.3

(i) One can also construct examples of *c*-weakly convex functions (for some $c>0$) that are not prox-convex, hence these two classes only contain some common elements without one of them being completely contained in the other.
(ii) While Examples 3.1 and 3.3 exhibit prox-convex functions that are also DC, the prox-convex functions presented in Example 3.2 are not DC. Examples of DC functions that are not prox-convex can be constructed as well, consequently, like in the case of *c*-weakly convex functions, these two classes only contain some common elements without one of them being completely contained in the other. Note moreover that different to the literature on algorithms for DC optimization problems (see, for instance, [2, 4]) where usually only critical points (and not optimal solutions) of such problems are determinable, for the DC functions that are also prox-convex proximal point methods are capable of delivering global minima (on the considered sets).
(iii) The remarkable properties of the Kurdyka-Łojasiewicz functions made them a standard tool when discussing proximal point type algorithms for nonconvex functions. As their definition requires proper closedness and the prox-convex functions presented in Example 3.2 are not closed, the class of prox-convex functions can be seen as broader in some sense than the one of the Kurdyka-Łojasiewicz ones. Similarly one can note that prox-convexity is not directly related to hypomonotonicity of subdifferentials (see [10, 18, 24], respectively).
(iv) At least due to the similar name, a legitimate question is whether the notion of prox-convexity is connected in any way with the prox-regularity (cf. [10, 20, 24]). While the latter asks a function to be locally lower semicontinuous around a given point, the notion we introduce in this work does not assume any topological properties on the involved function. Another difference with respect to this notion can be noticed in Sect. 4, where we show that the classical proximal point algorithm remains convergent towards a minimum of the function to be minimized even if this lacks convexity, but is prox-convex. On the other hand, the iterates of the modified versions of the proximal point method employed for minimizing prox-regular functions converge towards critical points of the latter. Last but not least note that, while in the mentioned works one uses tools specific to nonsmooth analysis such as generalized subdifferentials, in this paper we employ the convex subdifferential and some subdifferential notions specific to quasiconvex functions.

Necessary and sufficient hypotheses for condition (3.4) are given below.

#### Proposition 3.5

Let *K* be a closed set in $\mathbb {R}^{n}$ and $h: \mathbb {R}^{n} \rightarrow \overline{\mathbb {R}}$ be a proper, lower semicontinuous and prox-convex function such that $K \cap {{\,\mathrm{dom}\,}}\,h \ne \emptyset$. Let $\alpha > 0$ be the prox-convex value of *h* on *K*, and $z \in K$. Consider the following assertions

(*a*): $${{\,\mathrm{Prox}\,}}_{h} (K, z) = \{\overline{x}\}$$;
(*b*): $$z - \overline{x} \in \partial _{K} \left( \frac{1}{\alpha }h\right) (\overline{x})$$;
(*c*): $$(\frac{1}{\alpha }h)_{z} (\overline{x}) - (\frac{1}{\alpha }h)_{z} (x) \le - \frac{1}{2} \Vert x - \overline{x} \Vert ^{2}$$ for all $$x \in K$$;
(*d*): $$\overline{x} \in {{\,\mathrm{Prox}\,}}_{\frac{1}{\alpha }h} (K, z)$$.

Then

$$\begin{aligned} (a) ~ \Longrightarrow ~ (b) ~ \Longleftrightarrow ~ (c) ~ \Longrightarrow ~ (d). \end{aligned}$$

If $\alpha = 1$, then (*d*) implies (*a*) and all the statements are equivalent.

#### Proof

$(a) \Rightarrow (b)$: By definition of prox-convexity.

$$(b) \Leftrightarrow (c)$$: One has

3.9 $$\begin{aligned}&z - \overline{x} \in \partial _{K} ( \frac{1}{\alpha } h)(\overline{x}) ~ \Longleftrightarrow ~ (\frac{1}{\alpha } h) (\overline{x}) - (\frac{1}{\alpha } h)(x) \le \langle \overline{x} - z, x - \overline{x} \rangle , ~ \forall x \in K \nonumber \\&\quad \Longleftrightarrow ~ \frac{1}{\alpha } h(\overline{x}) - \frac{1}{\alpha } h(x) \le \frac{1}{2} \Vert z - x \Vert ^{2} - \frac{1}{2} \Vert z - \overline{x} \Vert ^{2} - \frac{1}{2} \Vert x - \overline{x} \Vert ^{2}, ~ \forall x \in K \nonumber \\&\quad \Longleftrightarrow ~ \frac{1}{\alpha }h(\overline{x}) + \frac{1}{2} \Vert z - \overline{x} \Vert ^{2} - \frac{1}{\alpha }h(x) - \frac{1}{2} \Vert z - x \Vert ^{2} \le - \frac{1}{2} \Vert x - \overline{x} \Vert ^{2}, ~ \forall x \in K \nonumber \\&\quad \Longleftrightarrow ~ (\frac{1}{\alpha } h)_{z} (\overline{x}) - (\frac{1}{ \alpha } h)_{z} (x) \le - \frac{1}{2} \Vert x - \overline{x} \Vert ^{2}, ~ \forall x \in K. \end{aligned}$$

$(c) \Rightarrow (d)$: As $- ({1}/{2}) \Vert x - \overline{x} \Vert ^{2} \le 0$ for all $x \in K$, (3.9) yields $\overline{x}\in {{\,\mathrm{Prox}\,}}_{(1/\alpha ) h} (K, z)$.

When $\alpha =1$, the implication $(d) \Rightarrow (a)$ is straightforward. $\square$

#### Remark 3.4

It follows from Proposition 3.5(*d*) that if *h* is prox-convex on *K* with prox-convex value $\alpha > 0$, then the function $(1/\alpha ) h$ is also prox-convex on *K* with prox-convex value 1. Moreover, ${{\,\mathrm{Prox}\,}}_{(1/\alpha ) h} = {{\,\mathrm{Prox}\,}}_{h}$.

If *h* is prox-convex with prox-convex value $\alpha$, then we know that ${{\,\mathrm{Prox}\,}}_{(1/\alpha ) h} = {{\,\mathrm{Prox}\,}}_{h}$ is a singleton, hence

3.10 $$\begin{aligned} ^{\frac{1}{\alpha }}h(z) = \min _{x \in K} \left( h(x) + \frac{\alpha }{2} \Vert z - x \Vert ^{2} \right) = h ({{{\,\mathrm{Prox}\,}}}_{h} (z)) + \frac{\alpha }{2} \Vert z - {{{\,\mathrm{Prox}\,}}}_{h}(z) \Vert . \end{aligned}$$

Consequently, $^{1/\alpha }h(z)\in \mathbb {R}$ for all $z\in \mathbb {R}^{n}$. Furthermore, we have the following statements.

#### Proposition 3.6

Let $h:\mathbb {R}^{n} \rightarrow \overline{\mathbb {R}}$ be proper, lower semicontinuous and prox-convex with prox-convex value $\alpha >0$ on a closed set $K \subseteq \mathbb {R}^{n}$ such that $K \cap {{\,\mathrm{dom}\,}}\,h \ne \emptyset$. Then $^{1/\alpha }h: \mathbb {R}^{n} \rightarrow \mathbb {R}$ is Fréchet differentiable everywhere and

3.11 $$\begin{aligned} \nabla \left( {^{\frac{1}{\alpha }}}h\right) =\alpha \left( {{\,\mathrm{Id}\,}}-{{{\,\mathrm{Prox}\,}}}_{\frac{1}{\alpha }h}\right) , \end{aligned}$$

is $\alpha$-Lipschitz continuous.

#### Proof

Let $x, y \in K$ with $x \ne y$. Set $\gamma = 1/\alpha$, $\overline{x} = {{\,\mathrm{Prox}\,}}_{h} (K, x)$ and $\overline{y} = {{\,\mathrm{Prox}\,}}_{h} (K, y)$. As *h* is prox-convex with prox-convex value $\alpha$, we have

$$\begin{aligned} h(z) - h(\overline{x}) \ge \alpha \langle x - \overline{x}, z - \overline{x} \rangle \, \forall \, z \in K \, \Longrightarrow \, h(\overline{y}) - h(\overline{x}) \ge \frac{1}{\gamma } \langle x - \overline{x}, \overline{y} - \overline{x} \rangle . \end{aligned}$$

From (2.17), we get

3.12 $$\begin{aligned} {}^{\gamma }h(y)-\,^{\gamma }h(x)&= h(\overline{y}) - h(\overline{x}) + \frac{1}{2\gamma } \left( \Vert y - \overline{y} \Vert ^{2} - \Vert x - \overline{x} \Vert ^{2}\right) \nonumber \\&\ge \frac{1}{2 \gamma } \left( 2 \langle x - \overline{x}, \overline{y} - \overline{x} \rangle + \Vert y - \overline{y} \Vert ^{2} - \Vert x - \overline{x} \Vert ^{2}\right) \nonumber \\&=\frac{1}{2 \gamma } \left( \Vert y - \overline{y} - x + \overline{x} \Vert ^{2} + 2 \langle y-x, x - \overline{x} \rangle \right) \nonumber \\&\ge \frac{1}{\gamma } \langle y - x, x - \overline{x} \rangle . \end{aligned}$$

Exchanging above *x* with *y* and $\overline{x}$ with $\overline{y}$, one gets

3.13 $$\begin{aligned} ^{\gamma }h(x) - \,^{\gamma }h(y) \ge \frac{1}{\gamma } \langle x - y, y - \overline{y} \rangle . \end{aligned}$$

It follows from equations (3.12) and (3.13) that

$$\begin{aligned} 0&\le \,^{\gamma }h(y) - \,^{\gamma } h(x) - \frac{1}{\gamma } \langle y - x, x - \overline{x} \rangle \\&\le - \frac{1}{\gamma } \langle x - y, y - \overline{y} \rangle - \frac{1}{\gamma } \langle y - x, x - \overline{x} \rangle \\&= \frac{1}{\gamma } \Vert y - x \Vert ^{2} + \frac{1}{\gamma } \langle y - x, \overline{x} - \overline{y} \rangle . \end{aligned}$$

As ${{\,\mathrm{Prox}\,}}_{K, h}$ is firmly nonexpansive on *K*, $\langle y - x, \overline{y} - \overline{x} \rangle \ge \Vert \overline{y} - \overline{x} \Vert ^{2} \ge 0$, then

$$\begin{aligned} 0&\le \,^{\gamma }h(y) - \,^{\gamma }h(x) - \frac{1}{\gamma } \langle y - x, x - \overline{x} \rangle \le \frac{1}{\gamma } \Vert y - x \Vert ^{2} \\&\Longrightarrow ~ \lim _{y \rightarrow x} \frac{^{\gamma }h(y) - \,^{\gamma }h(x) - \frac{1}{\gamma } \langle y - x, x - \overline{x} \rangle }{ \Vert y - x \Vert } = 0. \end{aligned}$$

Thus, $^{1/\alpha }h$ is Fréchet differentiable at every $x\in \mathbb {R}^{n}$, and $\nabla (^{1/\alpha }h)=\alpha ( {{\,\mathrm{Id}\,}}-$ ${{\,\mathrm{Prox}\,}}_{h})$. Since ${{\,\mathrm{Prox}\,}}_{h}$ is firmly nonexpansive, ${{\,\mathrm{Id}\,}}-{{\,\mathrm{Prox}\,}}_{h}$ is also firmly nonexpansive, so $\nabla (^{1/\alpha }h)$ is $\alpha$-Lipschitz continuous. $\square$

### Strongly G-subdifferentiable functions

Further we introduce and study a class of quasiconvex functions whose lower semicontinuous members are prox-convex.

#### Definition 3.2

Let *K* be a closed and convex set in $\mathbb {R}^{n}$ and $h: \mathbb {R}^{n} \rightarrow \overline{\mathbb {R}}$ be a proper and lower semicontinuous function such that $K \cap {{\,\mathrm{dom}\,}}\,h \ne \emptyset$. We call *h* strongly G-subdifferentiable on *K* if

(*a*): *h* is strongly quasiconvex on *K* for some $\beta \in [1, + \infty [$;
(*b*): for each $z \in K$ there exists $\overline{x}\in \mathbb {R}^{n}$ such that ${{\,\mathrm{Prox}\,}}_{h} (K, z) = \{\overline{x}\}$ and
  3.14 $$\begin{aligned} \frac{1}{2} (z - \overline{x}) \in \partial ^{\le }_{K} h(\overline{x}). \end{aligned}$$

Next we show that a lower semicontinuous and strongly G-subdifferentiable function on *K* is prox-convex.

#### Proposition 3.7

Let *K* be a closed and convex set in $\mathbb {R}^{n}$ and $h: \mathbb {R}^{n} \rightarrow \overline{\mathbb {R}}$ be a proper and lower semicontinuous function such that $K \cap {{\,\mathrm{dom}\,}}\,h \ne \emptyset$. If *h* is strongly G-subdifferentiable on *K*, then $h \in \Phi (K)$.

#### Proof

Let *h* be a lower semicontinuous and strongly G-subdifferentiable function. Then for every $z \in K$, there exists $\overline{x} \in K$ with $\overline{x} = {{\,\mathrm{Prox}\,}}_{h} (K, z)$. Hence, given any $y \in K$, we take $y_{\lambda } = \lambda y + (1 - \lambda ) \overline{x}$ with $\lambda \in [0, 1]$. Thus, by the definition of the proximity operator and the strong quasiconvexity of *h* on *K* for some $\beta \ge 1$, we have

$$\begin{aligned} h(\overline{x})&\le h(\lambda y + (1 - \lambda ) \overline{x}) + \frac{1}{2} \Vert \lambda y + (1 - \lambda ) \overline{x} - z \Vert ^{2} - \frac{1}{2} \Vert \overline{x} - z \Vert ^{2}\\&= h(\lambda y + (1 - \lambda ) \overline{x}) + \lambda \langle \overline{x} - z, y - \overline{x} \rangle + \lambda ^{2} \frac{1}{2} \Vert y - \overline{x} \Vert ^{2} \\&\le \max \{h(y), h(\overline{x})\} + \lambda \langle \overline{x} - z, y - \overline{x} \rangle + \frac{\lambda }{2}(\lambda \beta + \lambda - \beta ) \Vert y - \overline{x} \Vert ^{2}. \end{aligned}$$

We have two possible cases.

(*i*): If $$h(y) > h(\overline{x})$$, then
  $$\begin{aligned} h(\overline{x}) - h(y) \le \lambda \langle \overline{x} - z, y - \overline{x} \rangle + \frac{\lambda }{2}(\lambda \beta + \lambda - \beta ) \Vert y - \overline{x} \Vert ^{2}, \ \forall \lambda \in [0, 1]. \end{aligned}$$
  Hence, for $\lambda = 1/2$ and since $\beta \ge 1$, one has
  $$\begin{aligned} h(\overline{x}) - h(y)&\le \frac{1}{2} \langle \overline{x} - z, y - \overline{x} \rangle + \frac{1}{4} (\frac{1}{2} - \frac{\beta }{2}) \Vert y - \overline{x} \Vert ^{2} \\&\le \frac{1}{2} \langle \overline{x} - z, y - \overline{x} \rangle , \ \forall y \in K \backslash S_{h(\overline{x})} (h). \end{aligned}$$
(*ii*): If $h(y) \le h(\overline{x})$, then $y \in S_{h(\overline{x})} (h)$, it follows from Definition 3.2(*b*) that
  $$\begin{aligned} \frac{1}{2} (z - \overline{x}) \in \partial ^{\le }_{K} h(\overline{x}) \Longleftrightarrow h(\overline{x}) - h(y) \le \frac{1}{2} \langle \overline{x} - z, y - \overline{x} \rangle , \ \forall y \in K \cap S_{h(\overline{x})} (h). \end{aligned}$$
  Therefore, it follows that *h* satisfies (3.4) for $\alpha = {1}/{2}$, i.e., $h \in \Phi (K)$.

$$\square $$

#### Remark 3.5

(*i*): When $h:\mathbb {R}^{n}\rightarrow \overline{\mathbb {R}}$ is lower semicontinuous and strongly quasiconvex, as strongly quasiconvex functions are semistrictly quasiconvex, *h* is quasiconvex and every local minimum of *h* is a global minimum, too, so *h* is neatly quasiconvex, i.e., $\partial ^{<} h = \partial ^{\le } h$ (see [26, Proposition 9]). Therefore, we can replace $\partial ^{\le }_{K} h$ by $\partial ^{<}_{K} h$ in condition (3.14).
(*ii*): Strongly *G*-subdifferentiable functions are not necessarily convex as the function in Example 3.1 shows.

A family of prox-convex functions that are not strongly *G*-subdifferentiable can be found in Remark 3.6, see also Example 3.2.

Now, we study lower semicontinuous strongly quasiconvex functions for which the Gutierréz subdifferential is nonempty. To that end, we first recall the following definitions (adapted after [11, Definition 3.1]).

#### Definition 3.3

Let *K* be a nonempty set in $\mathbb {R}^{n}$ and $h: \mathbb {R}^{n} \rightarrow \overline{\mathbb {R}}$ with $K \cap {{\,\mathrm{dom}\,}}\,h \ne \emptyset$. We say that *h* is

(*a*): $\inf$*-compact* on *K* if for all $\overline{x} \in K$, $S_{h(\overline{x})} (h) \cap K$ is compact;
(*b*): $\alpha$*-quasiconvex* at $\overline{x} \in K$ $(\alpha \in \mathbb {R})$, if there exist $\rho > 0$ and $e \in \mathbb {R}^{n}$, $\Vert e \Vert = 1$, such that
  3.15 $$\begin{aligned} y \in K \cap \mathbb {B} (\overline{x}, \rho ) \cap S_{h(\overline{x})} (h) \Longrightarrow ~ \langle y - \overline{x}, e \rangle \ge \alpha \Vert y - \overline{x} \Vert ^{2}; \end{aligned}$$
(*c*): *positively quasiconvex* on *K* if for any $\overline{x}$ there exists $\alpha (\overline{x}) > 0$ such that *h* is $\alpha (\overline{x})$-quasiconvex on $S_{h(\overline{x})} (h)$.

The following result presents a connection between strongly quasiconvex functions and positively quasiconvex ones.

#### Proposition 3.8

Let $h: \mathbb {R}^{n} \rightarrow \mathbb {R}$ be a strongly quasiconvex function, $\overline{x} \in \mathbb {R}^{n}$ and $\alpha > 0$. Then the following assertions hold

(*a*): If $$\xi \in \partial \left( ({1}/{\alpha }) h\right) (\overline{x})$$, then
  3.16 $$\begin{aligned} \langle \xi , y - \overline{x} \rangle \le - \frac{\beta }{2 \alpha } \Vert y - \overline{x} \Vert ^{2}, ~ \forall y \in S_{h(\overline{x})} (h). \end{aligned}$$
(*b*): If $$\xi \in \partial ^{\le } h(\overline{x})$$, then
  3.17 $$\begin{aligned} \langle \xi , y - \overline{x} \rangle \le - \frac{\beta }{2} \Vert y - \overline{x} \Vert ^{2}, \ \forall y \in S_{h(\overline{x})} (h). \end{aligned}$$

As a consequence, in both cases, *h* is positively quasiconvex on $\mathbb {R}^{n}$.

#### Proof

The proofs are similar, so we only show (*a*). Take $\overline{x} \in \mathbb {R}^{n}$ and $\xi \in \partial \left( ({1}/{\alpha }) h\right) (\overline{x})$. Then,

$$\begin{aligned} \alpha \langle \xi , z - \overline{x} \rangle \le h(z) - h(\overline{x}), \ \forall z \in \mathbb {R}^{n}. \end{aligned}$$

Take $y \in S_{h(\overline{x})} (h)$ and $z = \lambda y + (1 - \lambda ) \overline{x}$ with $\lambda \in [0, 1]$. Then

$$\begin{aligned} \lambda \alpha \langle \xi , y - \overline{x} \rangle&\le h(\lambda y + (1 -\lambda ) \overline{x}) - h(\overline{x})\\&\le \max \{h(y), h(\overline{x})\} - \lambda (1 - \lambda ) \frac{\beta }{2}\Vert y - \overline{x} \Vert ^{2} - h(\overline{x})\\&= - \lambda (1 - \lambda ) \frac{\beta }{2} \Vert y - \overline{x} \Vert ^{2}. \end{aligned}$$

Then, for every $y \in S_{h(\overline{x})} (h)$, by dividing by $\lambda > 0$ and taking the limit when $\lambda$ descends towards 0, we have

$$\begin{aligned} \langle \xi , y - \overline{x} \rangle \le \lim _{\lambda \downarrow 0} \left( - (1 - \lambda ) \frac{\beta }{2 \alpha } \Vert y - \overline{x} \Vert ^{2}\right) = - \frac{\beta }{2\alpha } \Vert y - \overline{x} \Vert ^{2}. \end{aligned}$$

Now, since *h* is strongly quasiconvex, ${\arg \min }_{\mathbb {R}^{n}} h$ has at most one point. If $\overline{x} \in {\arg \min }_{\mathbb {R}^{n}} h$, then condition (3.15) holds immediately. If $\overline{x} \not \in {\arg \min }_{\mathbb {R}^{n}} h$, then $\xi \ne 0$, i.e., condition (3.15) holds for ${\beta }/{(2 \alpha \Vert \xi \Vert )} > 0$.

Therefore, *h* is positively quasiconvex on $\mathbb {R}^{n}$. $\square$

As a consequence, we have the following result.

#### Corollary 3.1

Let $h: \mathbb {R}^{n} \rightarrow \mathbb {R}$ be a lower semicontinuous and strongly quasiconvex function with $\beta = 1$, let $z \in \mathbb {R}^{n}$ and $\overline{x} \in {{\,\mathrm{Prox}\,}}_{h} (z)$. If there exists $\xi \in \partial ^{\le } h(\overline{x})$ such that

3.18 $$\begin{aligned} h_{z} (\overline{x}) - h_{z} (x) \le \langle \xi , y - \overline{x} \rangle , \ \forall y \in S_{h(\overline{x})} (h), \end{aligned}$$

then *h* is prox-convex on its sublevel set at the height $h(\overline{x})$, i.e., $h \in \Phi (S_{h(\overline{x})} (h))$.

#### Proof

If $\xi \in \partial ^{\le } h(\overline{x})$, and since *h* is lower semicontinuous and strongly quasiconvex with $\beta = 1$, then by Proposition 3.8(*b*), we have

$$\begin{aligned} h_{z} (\overline{x}) - h_{z} (x)&\le \langle \xi , y - \overline{x} \rangle \le - \frac{1}{2} \Vert y - \overline{x} \Vert ^{2}, \ \forall y \in S_{h(\overline{x})} (h), \\&\Longrightarrow ~ h(\overline{x}) - h(x) \le \frac{1}{2} \Vert z - y \Vert ^{2} - \frac{1}{2} \Vert z - \overline{x} \Vert ^{2} - \frac{1}{2} \Vert y - \overline{x} \Vert ^{2}, \ \forall y \in S_{h(\overline{x})} (h)\\&\Longleftrightarrow ~ h(\overline{x}) - h(x) \le \langle \overline{x} - z, x - \overline{x} \rangle , \ \forall y \in S_{h(\overline{x})} (h). \end{aligned}$$

Therefore, $$h \in \Phi (S_{h(\overline{x})} (h))$$. $$\square $$

Another consequence is the following sufficient condition for $\inf$-compactness under an *L*-Lipschitz assumption, which revisits [29, Corollary 1].

#### Corollary 3.2

Let $h: \mathbb {R}^{n} \rightarrow \mathbb {R}$ be an *L*-Lipschitz and strongly quasiconvex function. Then *h* is $\inf$-compact on $\mathbb {R}^{n}$.

#### Proof

If *h* is strongly quasiconvex, then *h* is neatly quasiconvex, and since *h* is *L*-Lipschitz, $\partial ^{\le } h (x) \ne \emptyset$ for all $x \in \mathbb {R}^{n}$ by Lemma 2.1(*b*). Now, by Proposition 3.8(*b*), it follows that *h* is positively quasiconvex on $\mathbb {R}^n$. Finally, *h* is $\inf$-compact on $\mathbb {R}^{n}$ by [11, Corollary 3.6]. $\square$

We finish this section with the following observation.

#### Remark 3.6

There are (classes of) prox-convex functions which are neither convex nor strongly quasiconvex. Indeed, for all $n \in \mathbb {N}$, we take $K_{n} := [-n, + \infty [$ and the continuous quasiconvex functions $h_{n}: K_{n} \rightarrow \mathbb {R}$ given by $h_{n} (x) = x^{3}$. Clearly, $h_{n}$ is neither convex nor strongly quasiconvex on $K_{n}$ hence also not strongly G-subdifferentiable either.

Take $n \in \mathbb {N}$. Then for all $z \in K_{n}$, ${\arg \min }_{K_{n}} h_{n} = {{\,\mathrm{Prox}\,}}_{h_{n}} (z) = \{-n\}$, thus $S_{h_{n} (\overline{x})} (h_{n}) = \{ \overline{x}\}$, i.e., $\partial ^{\le }_{K_{n}} h_{n} (\overline{x}) = \mathbb {R}^{n}$. Therefore, $h_{n} \in \Phi (K_{n})$ for all $n \in \mathbb {N}$. Taking also into consideration Corollary 3.1 one can conclude that the classes of strongly quasiconvex and prox-convex functions intersect without being included in one another.

#### Remark 3.7

All the prox-convex functions we have identified so far are semistrictly quasiconvex, too, while there are semistrictly quasiconvex functions that are not prox-convex (for instance $h:\mathbb {R}\rightarrow \mathbb {R}$ defined by $h(x) = 1$ if $x=0$ and $h(x) = 0$ if $x \ne 0$), hence the connection between the classes of prox-convex and semistrictly quasiconvex functions remains an open problem.

For a further study on strong quasiconvexity, positive quasiconvexity and $\inf$-compactness we refer to [11, 29, 30].

## Proximal point type algorithms for nonconvex problems

In this section we show (following the proof of [5, Theorem 28.1]) that the proximal point type algorithm remains convergent when the function to be minimized is proper, lower semicontinuous and prox-convex (on a given closed convex set), but not necessarily convex. Although the algorithm considered below is the simplest and most basic version available and some of the advances achieved in the convex case, such as accelerations and additional flexibility by employing additional parameters, are at the moment still open in the prox-convex setting, our investigations show that the proximal point type methods can be successfully extended towards other classes of nonconvex optimization problems.

### Theorem 4.1

Let *K* be a closed and convex set in $\mathbb {R}^{n}$ and $h: \mathbb {R}^{n} \rightarrow \overline{\mathbb {R}}$ be a proper, lower semicontinuous and prox-convex on *K* function such that ${\arg \min }_{K} h\ne \emptyset$ and $K \cap {{\,\mathrm{dom}\,}}\,h \ne \emptyset$. Then for any $k \in \mathbb {N}$, we set

4.1 $$\begin{aligned} x^{k+1} = {{{\,\mathrm{Prox}\,}}}_{h} (K, x^{k}) \end{aligned}$$

Then $\{x^{k}\}_k$ is a minimizing sequence of *h* over *K*, i.e., $h(x^{k}) \rightarrow \min _{x \in K} h(x)$ when $k\rightarrow +\infty$, and it converges to a minimum point of *h* over *K*.

### Proof

Since *h* is prox-convex on *K*, denote its prox-convex value by $\alpha > 0$ and for all $k \in \mathbb {N}$ one has

4.2 $$\begin{aligned} x^{k+1}&= {{{\,\mathrm{Prox}\,}}}_{h} (K, x^{k}) ~ \Longrightarrow ~ x^{k} - x^{k+1} \in \partial \left( \frac{1}{\alpha } h + \delta _K\right) (x^{k+1}) \nonumber \\&\Longleftrightarrow \alpha \langle x^{k} - x^{k+1}, x - x^{k+1} \rangle \le h(x) - h(x^{k+1}), \ \forall x \in K. \end{aligned}$$

Take $x = x^{k} \in K$, and since $\alpha > 0$, we have

4.3 $$\begin{aligned} 0 \le \langle x^{k} - x^{k+1}, x^{k} - x^{k+1} \rangle \le \frac{1}{\alpha } \left( h\left( x^{k}\right) - h\left( x^{k+1}\right) \right) , \end{aligned}$$

which yields $h(x^{k+1}) \le h(x^{k})$ for all $k \in \mathbb {N}$.

On the other hand, take $\overline{x} \in {\arg \min }_{K} h$. Then, for any $k \in \mathbb {N}$, by taking $x = \overline{x}$ in equation (4.2), we have

4.4 $$\begin{aligned} \Vert x^{k+1} - \overline{x} \Vert ^{2}&= \Vert x^{k+1} - x^{k} + x^{k} - \overline{x} \Vert ^{2} \nonumber \\&= \Vert x^{k+1} - x^{k} \Vert ^{2} + \Vert x^{k} - \overline{x} \Vert ^{2} + 2\langle x^{k+1} - x^{k}, x^{k} - \overline{x} \rangle \nonumber \\&= -\Vert x^{k+1} - x^{k} \Vert ^{2} + \Vert x^{k} - \overline{x} \Vert ^{2} + 2\langle x^{k+1} - x^{k}, x^{k+1} - \overline{x} \rangle \nonumber \\&\le \Vert x^{k} - \overline{x} \Vert ^{2} + \frac{2}{\alpha } \left( h(\overline{x}) - h\left( x^{k+1}\right) \right) \le \Vert x^{k} - \overline{x} \Vert ^{2}, \end{aligned}$$

where we used that $h(\overline{x}) \le h(x^{k+1})$. Thus, $\{x^{k} - \overline{x}\}_k$ is bounded. Then by [5, Theorem 28.1] $x^{k}$ converges to a point in ${\arg \min }_{K} h$ when $k \rightarrow + \infty$. Finally, since *h* is lower semicontinuous and *K* is closed, we have $\liminf _{k \rightarrow + \infty } h(x^{k}) = \min _{x \in K} h(x)$, which yields the conclusion by (4.3). $\square$

### Remark 4.1

From (4.4) one can deduce straightforwardly that the known ${\mathcal {O}}(1/n)$ rate of convergence of the proximal point algorithm holds in the prox-convex case, too.

### Remark 4.2

Although the function to be minimized in Theorem 4.1 by means of the proximal point algorithm is assumed to be prox-convex, its prox-convex value $\alpha > 0$ needs not be known, even if it plays a role in the proof.

### Remark 4.3

One can modify the proximal point algorithm by replacing in (4.1) the proximal step by ${{{\,\mathrm{Prox}\,}}}_{h} (S_{h(x^{k})} (h), x^{k})$ without affecting the convergence of the generated sequence. Note also that taking $K=\mathbb {R}^{n}$ in Theorem 4.1 one obtains the classical proximal point algorithm adapted for prox-convex functions and not for a restriction of such a function to a given closed convex set $K\subseteq \mathbb {R}^{n}$.

### Example 4.1

Let $K=[0, 2] \times \mathbb {R}$ and consider the function $h: K \rightarrow \mathbb {R}$ given by $h(x_{1}, x_{2}) = x^{2}_{2} - x^{2}_{1} - x_{1}$. Observe that *h* is strongly quasiconvex in the first argument, and convex and strongly quasiconvex in the second argument, hence *h* is strongly quasiconvex without being convex on *K*. Furthermore, by Example 3.1*h* is prox-convex on *K*. The global minimum of *h* over *K* is $(2, 0)^\top$ and it can be found by applying Theorem 4.1, i.e., via the proximal point algorithm, although the function *h* is not convex. First one determines the proximity operator

$$\begin{aligned} {{{\,\mathrm{Prox}\,}}}_h\left( K, \left( z_1, z_2\right) ^\top \right) = \left( \left\{ \begin{array}{cc} 0, &{} \text{ if } z_1\le -2 \\ 2, &{} \text{ if } z_1> -2 \end{array},\right. \frac{z_2}{3}\right) ^\top ,\ z_1, z_2\in \mathbb {R}. \end{aligned}$$

Taking into consideration the way *K* is defined, it follows that the proximal step in Theorem 4.1 delivers $x^{k+1} = (2, x^{k}_2/3)^\top$, where $x^k = (x^k_1, x^k_2)^\top$. Whatever feasible starting point $x^1 \in K$ of the algorithm is chosen, it delivers the global minimum of *h* over *K* because $x^k_1=2$ and $x^k_2=x^1_2/(3^{k-1})$ for all $k \in \mathbb {N}$.

## Conclusions and future work

We contribute to the discussion on the convergence of proximal point algorithms beyond convexity by introducing a new generalized convexity notion called *prox-convexity*. We identify some quasiconvex, weakly convex and DC functions (and not only) that satisfy the new definition and different useful properties of these functions are proven. Then we show that the classical proximal point algorithm remains convergent when the convexity of the proper lower semicontinuous function to be minimized is relaxed to prox-convexity (on a certain subset of the domain of the function).

In a future work, we aim to uncover more properties and develop calculus rules for prox-convex functions as well as to extend our investigation to nonconvex equilibrium problems and nonconvex mixed variational inequalities, to Hilbert spaces and to splitting methods, also employing Bregman distances instead of the classical one where possible.
